# CSF Proteomics Identifies Specific and Shared Pathways for Multiple Sclerosis Clinical Subtypes

**DOI:** 10.1371/journal.pone.0122045

**Published:** 2015-05-05

**Authors:** Timucin Avsar, İlknur Melis Durası, Uğur Uygunoğlu, Melih Tütüncü, Nuri Onat Demirci, Sabahattin Saip, O. Uğur Sezerman, Aksel Siva, Eda Tahir Turanlı

**Affiliations:** 1 Dr. Orhan Öcalgiray Molecular Biology-Biotechnology and Genetics Research Center, Istanbul Technical University, Istanbul, Turkey; 2 Faculty of Engineering & Natural Sciences, Biological Sciences & Bioengineering, Sabancı University, Istanbul, Turkey; 3 Department of Neurology, Cerrahpasa School of Medicine, Istanbul University, Istanbul, Turkey; 4 Molecular Biology and Genetics Department, Science and Letter Faculty, Istanbul Technical University, Istanbul, Turkey; 5 Medical Informatics Department, Acibadem University, Ataşehir, İstanbul, Turkey; Harbin Medical University, CHINA

## Abstract

Multiple sclerosis (MS) is an immune-mediated, neuro-inflammatory, demyelinating and neurodegenerative disease of the central nervous system (CNS) with a heterogeneous clinical presentation and course. There is a remarkable phenotypic heterogeneity in MS, and the molecular mechanisms underlying it remain unknown. We aimed to investigate further the etiopathogenesis related molecular pathways in subclinical types of MS using proteomic and bioinformatics approaches in cerebrospinal fluids of patients with clinically isolated syndrome, relapsing remitting MS and progressive MS (n=179). Comparison of disease groups with controls revealed a total of 151 proteins that are differentially expressed in clinically different MS subtypes. KEGG analysis using PANOGA tool revealed the disease related pathways including aldosterone-regulated sodium reabsorption (p=8.02x10^-5^) which is important in the immune cell migration, renin-angiotensin (p=6.88x10^-5^) system that induces Th17 dependent immunity, notch signaling (p=1.83x10^-10^) pathway indicating the activated remyelination and vitamin digestion and absorption pathways (p=1.73x10^-5^). An emerging theme from our studies is that whilst all MS clinical forms share common biological pathways, there are also clinical subtypes specific and pathophysiology related pathways which may have further therapeutic implications.

## Introduction

Multiple sclerosis (MS) is an immune-mediated neuro-inflammatory and neurodegenerative disease of the central nervous system (CNS) in which the major damage involves the myelin and axons [1, 2]. Although decades of research have been conducted, only recently immune-mediated and neurodegenerative processes of MS became more biologically and pathologically evident [3, 4]. It seems conceivable that in genetically susceptible individuals, release of CNS proteins into the periphery either due to an infection or other yet to be defined factor may triggers the loss of self-tolerance towards CNS proteins by the activation of myelin-reactive T cells [5–8]. Furthermore recent studies have highlightened a possible role for B cells in driving the pathologic immune response in MS [9]. Genome-wide association and candidate gene studies have identified variants in numerous genes including IL2R, IL7R, HLA-DRB regions [10, 11], but still complete understanding of their causation is lacking, not only because of the issues relating to individual changes having low functional significance, but also due to the complex nature of the disease which has low heritability [3, 12, 13]. Several proteomic studies have been published, mainly focusing on individual proteins for their biomarker potentiality for MS [14–16]. Even though these studies have shown a great variety of biomarker candidates, replications have been scarce probably due to the variations in the methodology, the type of tissues studied and the sample characteristics that were not extensively representative of the heterogeneous MS clinical spectrum [17, 18].

The complex nature of the disease is expressed as different clinical phenotypes within a continuum of disease status in which patients may remain in the early stages (CIS), intermediate stages (RRMS) or may progress to more severe forms (PPMS and SPMS) of this spectrum. Variation in the responses to therapy and different prognosis led many researchers to hypothesize that clinical types of MS may follow different pathologic pathways [19, 20]. Based on the clinical spectrum of the disease expression, one expects to see heterogeneity amongst different stages, but the current approaches are limited and show heterogeneity amongst patients belonging to not only different stages but also within the same clinical phenotype [4, 21, 22] To better understand the disease continuum, a novel systems approach involving analysis of available data in a pathway related context with PANOGA analysis tool, which is a pathway oriented analysis method bringing a systems level approach.

Here we aimed to analyze the CSF proteomic profile from a prospective cohort that has been followed for seven years, containing discrete clinical subtypes with a substantial sample size (N = 179). Our results revealed the presence of common disease pathways; renin angiotensin system and complement and coagulation cascade pathway shared by all disease subtypes. In our cohort of CIS patients’ upregulation of aldosterone pathway has been observed which correlates with recent findings of dysregulated salt metabolism in the development of MS [23]. We further obtained results for clinical subtype specific pathways such as vitamin digestion and absorption, NOD-Like receptor and Notch signaling pathways that showed correlation with pathology related clinical heterogeneity.

## Materials and Methods

### Patient selection

CSF was collected from patients undergoing lumbar puncture as part of their diagnostic workup at the Department of Neurology, Cerrahpaşa School of Medicine, Istanbul University. The samples comprised cerebrospinal fluid from 65 patients initially diagnosed with clinically isolated syndrome (CIS), 72 patients with relapsing remitting MS (RRMS), and 42 progressive MS (PMS) group which was compromised of 8 secondary progressive MS (SPMS) and 34 primary progressive (PPMS). Control samples included 22 patients wit h non-inflammatory neurologic disorders such as acute headache and pseudotumor cerebri and 20 other neurological diseases (OND) with neuro-inflammatory properties such as neuro-Behçet disease, neuro-sarcoidosis and polyneuropathies. All patients with CIS and MS were diagnosed initially according to McDonald 2005 [24] and Poser further evaluated based on the revised criteria of McDonald 2010 at the time of analysis.

In this study, CSF samples were collected prospectively, from all patients at the time of entry to the study. The study subjects were enrolled according to their clinical diagnosis, which were CIS, RRMS, PPMS, SPMS and control groups. After the enrolment each patient group were clinically followed prospectively. The CIS patients were treatment-naïve and endpoints for the CIS patients were having a second clinical attack or appearance or new MRI lesions on follow-up MRI studies fulfilling McDonald 2005 [24] criteria for the diagnosis of MS. In order to evaluate the difference between CIS patients remaining CIS and converting to clinically definite MS subtype, CIS group was separated into three different subgroups. MS suggestive CIS (MSs-CIS) indicates the CIS patients remaining CIS with a single clinical episode and MRI findings suggestive of MS but not fulfilling the diagnostic MRI criteria (either dissemination in time or space) to be diagnosed as MS during the follow up period. Single attack MS (SA-MS) indicates the patients who fulfill 2010 McDonald Criteria either at onset of clinical episode or during the follow up period. Clinically definite MS (CDMS) patients indicates the patients who fulfills the McDonald 2010 by having a second clinical attack previously Poser criteria [25]. RRMS patient were followed for any change in their disability levels by EDSS and composite index and whether they would convert to SPMS or not. Single attack progressive MS (SAP-MS) patients were not included in the study. On the other hand, for the CIS group, serum and CSF samples were collected within the first 10 days of neurological episode of CIS and prior to any treatment. The patients then were clinically followed prospectively until the termination of the study. Additionally, CIS patients were followed for the first 18 months both by clinical and imaging examination every 6 months and then on yearly basis or they were seen whenever they had a new clinical symptom and a sign suggestive of an MS episode.

**Fig 1:**
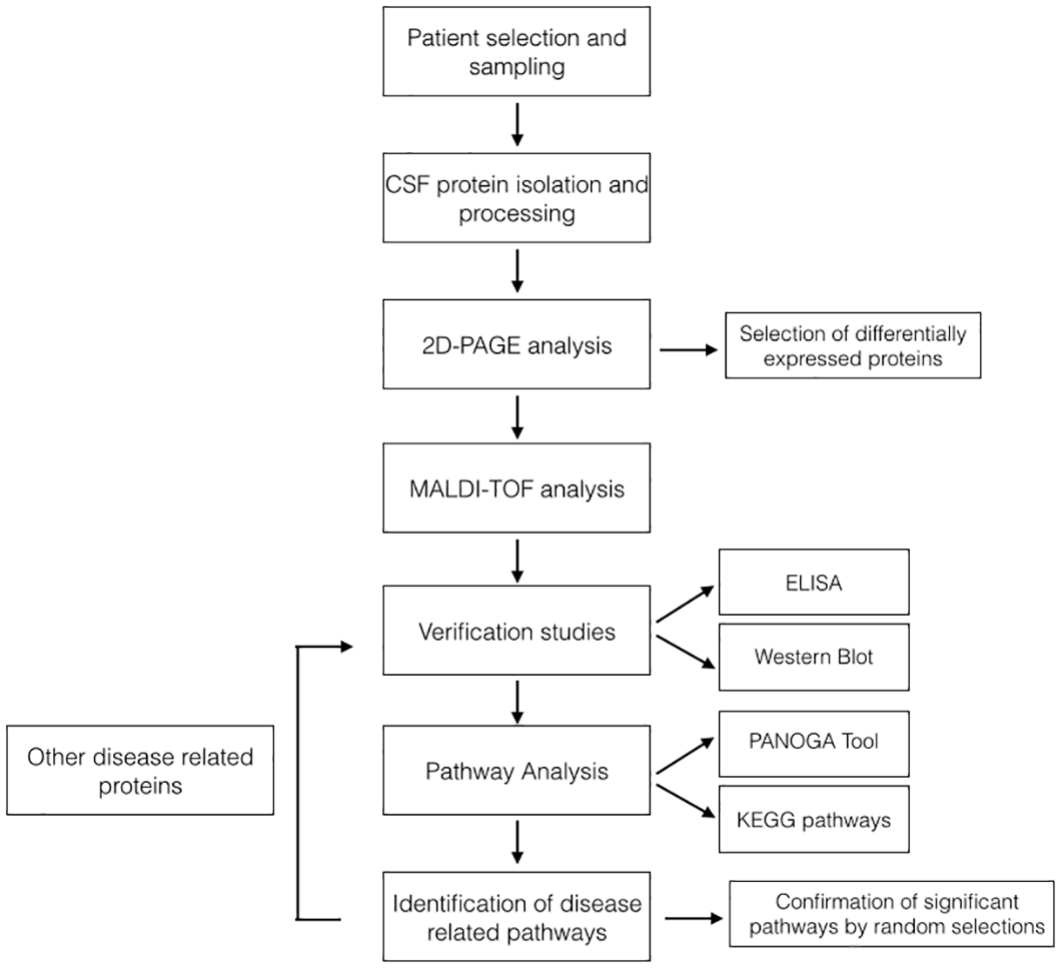
Overview and design of the study. This flowchart summarizes our approach in identification of MS related molecular pathways using a combination of two-dimensional gel electrophoresis and mass spectroscopy, with bioinformatics analysis.

The summarized clinical and demographic characteristics of patients and controls are shown in Table 1. All technical and analytical procedures were summarized in Fig 1. The CSF samples were collected and stored according to the guidelines from the BioMSeu consortium [26] Immediately after sampling, CSF was then centrifuged at 16,000 g (48°C) for 10 min to eliminate cells and other insoluble materials. The concentration of total protein of each sample was measured by Bradford assay using BSA as standard (Protein Assay Kit, Bio-Rad, Hercules, CA) after centrifugation. Then samples were stored at -80°C before the succeeding manipulation. The study was approved by ethical committee of The Istanbul University, Cerrahpaşa School of Medicine. Written informed consent was obtained from all patients and controls.

**Table 1:** Clinical and demographical data of study group.

	Age	Age at onset	Gender (F:M)	Disease duration* (Months)	EDSS	Follow up period ** (Months)	CSF IgG Index
**CIS (65)**	33.4 ± 6.2	30.2 ± 6.0	43:22	32.31 ± 9.2	1.13 ± 0.3	31.7 ± 5.8	1.12 ± 0.4
**MSs-CIS (35)**	31.8 ± 6.4	28.6 ± 5.8	20:12	30.81 ± 9.4	1.20 ± 0.4	30.12 ± 5.2	1.08 ± 0.3
**SA-MS (30)**	35.3 ± 7.1	32.2 ± 6.3	23:10	33.60 ± 8.1	1.05 ± 0.1	33.54 ± 6.2	1.16 ± 0.6
**RRMS (72)**	34.50 ± 6.3	28.91 ± 5.2	46:26	52.4 ± 9.2	3.3 ± 1.2	46.4 ± 8.4	1.19 ± 0.7
**PMS** *** **(42)**	44.2 ± 8.1	33.1 ± 7.5	25:17	70.6 ± 11.4	6.2 ± 2.7	60.1 ± 9.5	1.51 ± 0.9
**NonMS Control (22)**	32.7 ± 3.3	-	16:6	-	-	-	-
**OND (20)**	38.3 ± 4.7	-	14:6	-	-	-	-

* The time between first clinical attack and last examination

** The time between first and last examination

*** PMS samples were obtained from our biobank

Study groups with case and control samples were summarized in terms of demographical and clinical information. Age at analysis, age of disease onset, gender, expanded disability status scale (EDSS), disease duration which indicates the time between first clinical attack and last examination, follow-up period which indicates the time between first and last examination and finally CSF IgG index, which is calculated as IgG to CSF albumin ratio compared to the serum IgG to serum albumin ratio, were summarized. NonMS control samples consisted of acute headache and pseudotumour cerebri patients whereas, other neurological disease (OND) consisted of neuro-Behçet disease, neuro-sarcoidosis and polyneuropathies.

### Sample depletion and preparation

After thawing, CSF samples were first concentrated and then albumin and IgG depleted with the ProteoPrep Immunoaffinity Albumin and IgG Depletion Kit (Sigma-Aldrich, St. Louis, Missouri, USA) following the manufacturer’s recommendations. Subsequently, samples were precipitated by adding four volumes of ice-cold acetone overnight, centrifuged and the protein pellet diluted in water. Finally, protein concentration was determined using the Bradford’s protein quantification method (Bio-Rad Protein Assay, Bio-Rad Laboratories GmbH, Munich, Germany).

### 2DE-PAGE and Mass Spectrophotometry

**Fig 2:**
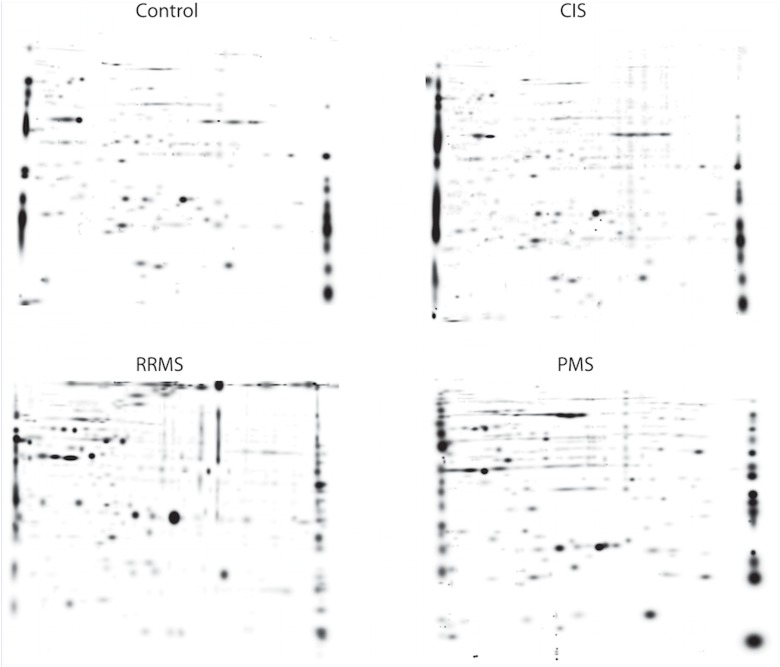
Representative 2D-PAGE image of each study group including control, CIS, RRMS and PMS.

Total protein concentrations were equalized, to adjust different protein concentrations in the samples. All samples were prepared and analyzed by 2DE in a blinded and randomized sequence and the total protein concentration loaded was 600 μg/gel. Isoelectric focusing was performed using 17 cm IPG strips pH 4-7L (Bio-Rad), and the second dimension separation was conducted using 1.0×220×200mm 12.5%TSDS-PAGE. 2D-gels were stained with Sypro Ruby (Molecular Probes, USA) over night and finally scanned at 100 μm resolution (ChemiDoc MP Imaging System, Bio-Rad, Hercules, CA, USA). Image files were processed using the PDQuest software (version 7.3, Bio-Rad, Hercules, CA, USA). Detected protein spots were then matched between gels and a synthetic master image was prepared to represent a majority of the protein spots present in all gels belonging to control samples. Matching rate of the spot analysis was evaluated by comparison of each sample gel with the master gel. Spots corresponding to the same spots in both gels indicate the matching rate. Determination of matching rate maintains the efficacy of the comparison in terms of the variation. The higher match rate provides better comparison. Spot identification was followed by image analysis and quantification (relative integrated optical density of protein spots) using PDQuest software. Representative figure of 2D-PAGE images for each groups were shown in Fig 2. Protein spots of interest were excised from gels and transferred to 96-well plates. Interested proteins were selected based on the comparisons of control groups including healthy control group and other neurological subject groups (OND). At least 2 times differentially expressed proteins (increased or decreased) were selected. Proteins were digested with trypsin and the samples were further purified and MALDI-TOF/MS was performed. Results of MALDI-TOF analysis for each individual, with the fold changes were given as supplementary file (S1 Data).

### Pathway analysis

For each patient in each MS group (CIS, RRMS, PMS) mean protein fold change values were calculated. T-test Statistics is applied for each group to identify significantly changing proteins (p value obtained) in that group compared to two different control groups. The lists of significant genes along with their p values are given as input to PANOGA to identify pathways with proteins that are significantly altered for each MS group.

PANOGA first searches out active sub-networks containing most of the disease affected proteins in the human PPI network. We used Goh et al.’s human PPI network in this study [27]. jActive Modules algorithm [28] is employed to identify the sub-networks taking into account the P-values of each gene with the network topology to extract potentially meaningful active sub-networks that overlaps at most 50% with each other.

The next step following the identification of sub-networks is to evaluate whether these sub-networks are biologically meaningful. For each sub-network, PANOGA computes the number of the genes in an identified sub-network that are also found in a specific human biochemical pathway, compared to the overall number of genes described for that pathway. In this functional enrichment step, PANOGA uses a two-sided (Enrichment/Depletion) test based on the hypergeometric distribution to examine the association between MS-related genes and the genes in each KEGG pathway. To correct the p-values for multiple testing, the Bonferroni correction procedure was applied on the p-values of each identified pathway. If a KEGG pathway is determined statistically significant for at least one of the active sub-networks, PANOGA adds this pathway into our final list of significant KEGG pathways associated with disease. If a pathway appears in more than one subnetwork analysis only the most significant one is reported. The details of these steps can be found in Gungor BB. et al. studies [29–33]. Statistical significance of the pathways in relation to MS groups is tested by shuffling the MS groups randomly 10 times and selecting CIS, PPMS and RRMS patients from each shuffled patient data. Then PANOGA is employed for each random MS group to identify significant pathway. The results of the random patient data is summarized in S1 Table.

### Immuno-assays for verification of differentially abundant proteins

For seven proteins (Apolipoprotein E, Apolipoprotein A-IV, Prostaglandin D2 Synthase, Transthyretin, Alpha-2 Macroglobulin, Anti-Trypsin and Vitamin D Binding Protein), which were found differentially expressed among MS subtypes. We performed validation experiments via commercially available ELISA kits and by western blot. The samples of the verification cohort were the same samples with proteome studies. For ELISA studies Blue Gene (Shangai Biotech) commercial kits were used according to manufacturers specifications. For western blot verifications Santa Cruz Biotenchnology Inc. (USA) antibodies were used. We performed a two-step western blot using primary goat antibodies and secondary anti-goat antibodies. Protein transfer was checked by Ponceau staining. Quantitative assessment of the gel bands after photoluminescence was performed using Image J software.

### Statistical analysis

Normalized volumes of matched spots were exported into Graphpad software v.4.0 for statistical evaluation. ANOVA test with Bonferroni correction was used to perform pair-wise comparisons between the cohorts in order to find spots with significantly different expression. MS/MS analysis was performed on polypeptide spots with an expression threshold higher than 2 and was significant with P < 0.05.

## Results

An average of 982 ± 34 protein spots were detected in the CSF samples from MS and control patients. Analysis of these spots revealed 382 spots with more than 2-fold difference in expression levels between samples and control master gel. A two- fold difference for the comparison was an selected based on the optimum spot difference and previous literature findings [34]. Variability of spot abundance within groups was estimated using coefficient of variation (CV). Average CV of global matching was about 24.21%, intra-group CV was about 23% for control, 26% for CIS, 22% for PMS and 22% for RRMS groups.

The difference was assessed by the independent samples t-test (P<0.05). The identification of selected spots was accomplished by MALDI-TOF/MS analysis and database searched in Swiss-Prot showed 151 distinct proteins corresponded.

### Common pathways for all disease subtypes

KEGG pathway analysis using the gene annotations of corresponding proteins present in the samples and their level of change, revealed the abundance of biological pathways. Comparison of disease subtypes with the total control group indicated that the renin-angiotensin system (RAS) and complement and coagulation cascade (CCC) pathways were common in all disease subtypes, with varying significances (given in Tables 2, 3, 4). Pathway associated proteins found in subnetworks were the angiotensin and MAS1 proteins. Mean level of angiotensin protein in the CIS group was increased 4.2 folds, 3.5 fold in RRMS group and 2.8 in PMS group compared to total control group (p<0.05). For complement and coagulation cascade pathway, associated proteins were Kininogen 1, Complement Component 7, Alpha-2-Macroglobulin, Serpin E1, Serpin G1, Serpin A1, Plasminogen proteins. Among them, Serpin E1 and Kininogen were decreased, whereas alpha-2-Macroglobulin levels were increased compared to control groups.

**Table 2:** Molecular pathways and associated proteins in CIS subtype.

KEGG ID	KEGG Term	Term P values Corr Bonf	Times Found	Pathway Assoc. Genes Found in Subnetworks	Pathway Associated Genes Not Found in Subnetworks
KEGG:04614	Renin-angiotensin system	6,88E-06	5	AGT, MAS1	ACE, ACE2, AGTR1, AGTR2, ANPEP, CMA1, CPA3, CTSA, CTSG, ENPEP, LNPEP, MME, NLN, REN, THOP1
KEGG:03050	Proteasome	4,32E-08	1	PSMB10, PSMA2, PSMA1, PSMD13, PSMB2, PSME3	IFNG, POMP, PSMA3, PSMA4, PSMA5, PSMA6, PSMA7, PSMA8, PSMB1, PSMB11, PSMB3, PSMB4, PSMB5, PSMB6, PSMB7, PSMB8, PSMB9, PSMC1, PSMC2, PSMC3, PSMC4, PSMC5, PSMC6, PSMD1, PSMD11, PSMD12, PSMD14, PSMD2, PSMD3, PSMD4, PSMD6, PSMD7, PSMD8, PSME1, PSME2, PSME4, PSMF1, SHFM1
KEGG:04610	Complement and coagulation cascades	4,19E-09	2	KNG1, A2M, SERPINE1, F2, SERPING1, SERPINA1, PLG	BDKRB1, BDKRB2, C1QA, C1QB, C1QC, C1R, C1S, C2, C3, C3AR1, C4A, C4B, C4BPA, C4BPB, C5, C5AR1, C6, C7, C8A, C8B, C8G, C9, CD46, CD55, CD59, CFB, CFD, CFH, CFI, CPB2, CR1, CR2, F10, F11, F12, F13A1, F13B, F2R, F3, F5, F7, F8, F9, FGA, FGB, FGG, KLKB1, MASP1, MASP2, MBL2, PLAT, PLAU, PLAUR, PROC, PROS1, SERPINA5, SERPINC1, SERPIND1, SERPINF2, TFPI, THBD, VWF
KEGG:05020	Prion diseases	9,05E-10	1	C8A, C8B, C9, C8G	BAX, C1QA, C1QB, C1QC, C5, C6, C7, CCL5, EGR1, ELK1, FYN, HSPA1A, HSPA5, IL1A, IL1B, IL6, LAMC1, LOC100506742, MAP2K1, MAP2K2, MAPK1, MAPK3, NCAM1, NCAM2, NOTCH1, PRKACA, PRKACB, PRKACG, PRKX, PRNP, SOD1, STIP1

Molecular pathways and associated proteins in CIS subtype were shown. Individual protein list of each CIS patients were processed and compared with control samples regarding their fold changes. Table shows the targeted KEGG pathways with the database ID and names. P values were given with the Bonferroni Corrections. ‘Times found’ indicates the co-occurrence of target proteins in subnetworks. Other pathway-associated proteins that are not found in subnetworks were also listed. Regarding the common and shared pathways, proteasome pathway is only found in CIS subtype.

**Table 3:** Molecular pathways and associated proteins in RRMS subtype.

KEGG ID	KEGG Term	Term P values Corr Bonf	Times Found	Pathway Assoc. Genes Found in Subnetworks	Pathway Associated Genes Not Found in Subnetworks
KEGG:04614	Renin-angiotensin system	6,88E-06	4	AGT, MAS1	ACE, ACE2, AGTR1, AGTR2, ANPEP, CMA1, CPA3, CTSA, CTSG, ENPEP, LNPEP, MME, NLN, REN, THOP1
KEGG:04977	Vitamin digestion and absorption	1,73E-05	1	APOB, CUBN, APOA1	ABCC1, APOA4, APOB, AWAT2, BTD, C20orf54, FOLH1, GIF, LMBRD1, LRAT, MMACHC, PLB1, PNLIP, RBP2, SLC19A1, SLC19A2, SLC19A3, SLC23A1, SLC46A1, SLC5A6, TCN2
KEGG:04330	Notch signaling pathway	3,89E-10	2	NOTCH3, NCSTN, NOTCH2, APH1A, RBPJ	ADAM17, CIR1, CREBBP, CTBP1, CTBP2, DLL1, DLL3, DLL4, DTX1, DTX2, DTX3, DTX3L, DTX4, DVL1, DVL2, DVL3, EP300, HDAC1, HDAC2, HES1, HES5, JAG1, JAG2, KAT2A, KAT2B, LFNG, MAML1, MAML2, MAML3, MFNG, NCOR2, NOTCH1, NOTCH4, NUMB, NUMBL, PSEN1, PSEN2, PSENEN, PTCRA, RBPJL, RFNG, SNW1
KEGG:04610	Complement and coagulation cascades	2,10E-09	1	KNG1, A2M, SERPINE1, F2, SERPING1, SERPINA1, PLG	BDKRB1, BDKRB2, C1QA, C1QB, C1QC, C1R, C1S, C2, C3, C3AR1, C4A, C4B, C4BPA, C4BPB, C5, C5AR1, C6, C7, C8A, C8B, C8G, C9, CD46, CD55, CD59, CFB, CFD, CFH, CFI, CPB2, CR1, CR2, F10, F11, F12, F13A1, F13B, F2R, F3, F5, F7, F8, F9, FGA, FGB, FGG, KLKB1, MASP1, MASP2, MBL2, PLAT, PLAU, PLAUR, PROC, PROS1, SERPINA5, SERPINC1, SERPIND1, SERPINF2, TFPI, THBD, VWF
KEGG:05130	Pathogenic Escherichia coli infection	2,12E-05	1	ACTB, KRT18, FYN, TUBA4A, NCL, CTNNB1	ABL1, ACTG1, ARHGEF2, ARPC1A, ARPC1B, ARPC2, ARPC3, ARPC4, ARPC5, ARPC5L, CD14, CDC42, CDH1, CLDN1, CTTN, EZR, HCLS1, ITGB1, LOC100506658, LY96, NCK1, NCK2, PRKCA, RHOA, ROCK1, ROCK2, TLR4, TLR5, TUBA1A, TUBA1B, TUBA1C, TUBA3C, TUBA3D, TUBA3E, TUBA8, TUBAL3, TUBB, TUBB1, TUBB2A, TUBB2B, TUBB2C, TUBB3, TUBB4, TUBB6, TUBB8, WAS, WASL, YWHAQ, YWHAZ
KEGG:05020	Prion diseases	9,05E-10	2	C8A, C8B, C9, C8G	BAX, C1QA, C1QB, C1QC, C5, C6, C7, CCL5, EGR1, ELK1, FYN, HSPA1A, HSPA5, IL1A, IL1B, IL6, LAMC1, LOC100506742, MAP2K1, MAP2K2, MAPK1, MAPK3, NCAM1, NCAM2, NOTCH1, PRKACA, PRKACB, PRKACG, PRKX, PRNP, SOD1, STIP1

Molecular pathways and associated proteins in RRMS subtype were shown. Individual protein list of each RRMS patients were processed and compared with control samples regarding their fold changes. Table shows the targeted KEGG pathways with the database ID and names. P values were given with the Bonferroni Corrections. ‘Times found’ indicates the co-occurrence of target proteins in subnetworks. Other pathway-associated proteins that are not found in subnetworks were also listed. Regarding the common and shared pathways, Pathogenic Escherichia coli infection pathway is only found in RRMS subtype.

**Table 4:** Molecular pathways and associated proteins in PMS subtype.

KEGG ID	KEGG Term	Term P values Corr Bonf	Times Found	Pathway Assoc. Genes Found in Subnetworks	Pathway Associated Genes Not Found in Subnetworks
KEGG:04614	*Renin-angiotensin system*	6,88E-06	5	AGT, MAS1	ACE, ACE2, AGTR1, AGTR2, ANPEP, CMA1, CPA3, CTSA, CTSG, ENPEP, LNPEP, MME, NLN, REN, THOP1
KEGG:04977	Vitamin digestion and absorption	2,7E-06	1	APOA4, APOA1, SCARB1	ABCC1, APOB, AWAT2, BTD, C20orf54, CUBN, FOLH1, GIF, LMBRD1, LRAT, MMACHC, PLB1, PNLIP, RBP2, SLC19A1, SLC19A2, SLC19A3, SLC23A1, SLC46A1, SLC5A6, TCN2
KEGG:04330	Notch signaling pathway	8,9E-07	1	NOTCH2, EP300, PSEN1, DTX1, JAG2	ADAM17, APH1A, CIR1, CREBBP, CTBP1, CTBP2, DLL1, DLL3, DLL4, DTX2, DTX3, DTX3L, DTX4, DVL1, DVL2, DVL3, HDAC1, HDAC2, HES1, HES5, JAG1, KAT2A, KAT2B, LFNG, MAML1, MAML2, MAML3, MFNG, NCOR2, NCSTN, NOTCH1, NOTCH3, NOTCH4, NUMB, NUMBL, PSEN2, PSENEN, PTCRA, RBPJ, RBPJL, RFNG, SNW1
KEGG:04610	*Complement and coagulation cascades*	3,9E-09	1	KNG1, F12, A2M, F2, SERPING1, SERPINA1, PLG	BDKRB1, BDKRB2, C1QA, C1QB, C1QC, C1R, C1S, C2, C3, C3AR1, C4A, C4B, C4BPA, C4BPB, C5, C5AR1, C6, C7, C8A, C8B, C8G, C9, CD46, CD55, CD59, CFB, CFD, CFH, CFI, CPB2, CR1, CR2, F10, F11, F13A1, F13B, F2R, F3, F5, F7, F8, F9, FGA, FGB, FGG, KLKB1, MASP1, MASP2, MBL2, PLAT, PLAU, PLAUR, PROC, PROS1, SERPINA5, SERPINC1, SERPIND1, SERPINE1, SERPINF2, TFPI, THBD, VWF

Molecular pathways and associated proteins in PMS subtype were shown. Individual protein list of each PMS patients were processed and compared with control samples regarding their fold changes. Table shows the targeted KEGG pathways with the database ID and names. P values were given with the Bonferroni Corrections. ‘Times found’ indicates the co-occurrence of target proteins in subnetworks. Other pathway-associated proteins that are not found in subnetworks were also listed.

### Subtype specific pathways

Besides common pathways, bioinformatic analysis also revealed the subtype specific and shared pathways between different subtypes. PMS and RRMS groups showed an affected vitamin digestion and absorption system (p = 2.7x10^-6^, p = 1.73x10^-5^), and notch signaling pathways (p = 8,9x10^-7^, p = 3.89x10^-10^). CIS and RRMS groups shared activated prion disease pathway (p = 9.05x10^-10^, 9.05x10-^10^), which is related with neuronal degeneration. Besides shared subtypes, RRMS group showed activated pathogenic *E*.*coli* absorption pathway (p = 2.12x10^-5^), indicating the importance of inflammatory cell movement. Subgroup analysis comprising CIS patients revealed differences between MSs-CIS, SA-MS and CDMS subgroups, defined according to McDonald 2010 diagnostic criteria. SA-MS group showed specific effects in the notch signaling pathway (p = 3.89x10^-10^), the Type II diabetes mellitus pathway (p = 7.73x10^-10^) and the aldosterone regulated sodium reabsorption pathway (1.78x10^-4^). Additionally, involvement of NOD-like receptor signaling pathway in SA-MS group (p = 6.69x10^-8^), and prion disease pathway (p = 1.45x10^-10^) and pathogenic E.coli infection pathways (p = 1.61x10^-10^) in CDMS group were observed. Proteins showing significant variation in diseased individuals that are present in these pathways are given in Table 5.

**Table 5:** Molecular pathways and associated proteins revealed by CIS subgroup analysis.

MSs-CIS					
KEGG ID	KEGG Term	Term P values Corr Bonf	Times Found	Pathway Associated Genes Found in Subnetworks	Pathway Associated Genes Not Found in Subnetworks
KEGG:04930	Type II diabetes mellitus	7,73E-07	1	IRS4, MAPK1, IRS2, INSR, PIK3R1, PIK3R2	ABCC8, ADIPOQ, CACNA1A, CACNA1B, CACNA1C, CACNA1D, CACNA1E, CACNA1G, GCK, HK1, HK2, HK3, HKDC1, IKBKB, INS, IRS1, KCNJ11, MAFA, MAPK10, MAPK3, MAPK8, MAPK9, MTOR, PDX1, PIK3CA, PIK3CB, PIK3CD, PIK3CG, PIK3R3, PIK3R5, PKLR, PKM2, PRKCD, PRKCE, PRKCZ, SLC2A2, SLC2A4, SOCS1, SOCS2, SOCS3, SOCS4, TNF
KEGG:04614	*Renin-angiotensin system*	6,88E-06	4	AGT, MAS1	ACE, ACE2, AGTR1, AGTR2, ANPEP, CMA1, CPA3, CTSA, CTSG, ENPEP, LNPEP, MME, NLN, REN, THOP1
KEGG:04330	Notch signaling pathway	3,89E-10	1	NOTCH3, NCSTN, NOTCH2, APH1A, RBPJ	ADAM17, CIR1, CREBBP, CTBP1, CTBP2, DLL1, DLL3, DLL4, DTX1, DTX2, DTX3, DTX3L, DTX4, DVL1, DVL2, DVL3, EP300, HDAC1, HDAC2, HES1, HES5, JAG1, JAG2, KAT2A, KAT2B, LFNG, MAML1, MAML2, MAML3, MFNG, NCOR2, NOTCH1, NOTCH4, NUMB, NUMBL, PSEN1, PSEN2, PSENEN, PTCRA, RBPJL, RFNG, SNW1
KEGG:04610	*Complement and coagulation cascades*	1,05E-08	1	KNG1, C7, A2M, SERPINE1, SERPING1, SERPINA1, PLG	BDKRB1, BDKRB2, C1QA, C1QB, C1QC, C1R, C1S, C2, C3, C3AR1, C4A, C4B, C4BPA, C4BPB, C5, C5AR1, C6, C8A, C8B, C8G, C9, CD46, CD55, CD59, CFB, CFD, CFH, CFI, CPB2, CR1, CR2, F10, F11, F12, F13A1, F13B, F2, F2R, F3, F5, F7, F8, F9, FGA, FGB, FGG, KLKB1, MASP1, MASP2, MBL2, PLAT, PLAU, PLAUR, PROC, PROS1, SERPINA5, SERPINC1, SERPIND1, SERPINF2, TFPI, THBD, VWF
KEGG:04960	Aldosterone-regulated sodium reabsorption	1,78E-04	1	MAPK1, INSR, PIK3R1, PIK3R2	ATP1A1, ATP1A2, ATP1A3, ATP1A4, ATP1B1, ATP1B2, ATP1B3, ATP1B4, FXYD2, FXYD4, HSD11B2, IGF1, INS, IRS1, KCNJ1, KRAS, MAPK3, NEDD4L, NR3C2, PDPK1, PIK3CA, PIK3CB, PIK3CD, PIK3CG, PIK3R3, PIK3R5, PRKCA, PRKCB, PRKCG, SCNN1A, SCNN1B, SCNN1G, SFN, SGK1, SLC9A3R2
**SA-MS**					
KEGG:04621	NOD-like receptor signaling pathway	6,69E-08	1	HSP90AA1, RELA, NFKBIA, TRAF6, TNFAIP3, IKBKB	BIRC2, BIRC3, CARD18, CARD6, CARD8, CARD9, CASP1, CASP5, CASP8, CCL2, CCL5, CHUK, CXCL1, CXCL2, ERBB2IP, HSP90AB1, HSP90B1, IKBKG, IL18, IL1B, IL6, IL8, MAP3K7, MAPK1, MAPK10, MAPK11, MAPK12, MAPK13, MAPK14, MAPK3, MAPK8, MAPK9, MEFV, NAIP, NFKB1, NFKBIB, NLRC4, NLRP1, NLRP3, NOD1, NOD2, PSTPIP1, PYCARD, PYDC1, RIPK2, SUGT1, TAB1, TAB2, TAB3, TNF, TRIP6
KEGG:04614	*Renin-angiotensin system*	6,88E-09	7	AGT, MAS1	ACE, ACE2, AGTR1, AGTR2, ANPEP, CMA1, CPA3, CTSA, CTSG, ENPEP, LNPEP, MME, NLN, REN, THOP1
KEGG:04610	*Complement and coagulation cascades*	7,89E-02	2	A2M, THBD, F5, F2, SERPING1, SERPINA1, F7, PROC	BDKRB1, BDKRB2, C1QA, C1QB, C1QC, C1R, C1S, C2, C3, C3AR1, C4A, C4B, C4BPA, C4BPB, C5, C5AR1, C6, C7, C8A, C8B, C8G, C9, CD46, CD55, CD59, CFB, CFD, CFH, CFI, CPB2, CR1, CR2, F10, F11, F12, F13A1, F13B, F2R, F3, F8, F9, FGA, FGB, FGG, KLKB1, KNG1, MASP1, MASP2, MBL2, PLAT, PLAU, PLAUR, PLG, PROS1, SERPINA5, SERPINC1, SERPIND1, SERPINE1, SERPINF2, TFPI, VWF
**CDMS**					
KEGG:04610	*Complement and coagulation cascades*	3,80E-07	1	KNG1, F12, A2M, F2, SERPING1, SERPINA1, PLG	BDKRB1, BDKRB2, C1QA, C1QB, C1QC, C1R, C1S, C2, C3, C3AR1, C4A, C4B, C4BPA, C4BPB, C5, C5AR1, C6, C7, C8A, C8B, C8G, C9, CD46, CD55, CD59, CFB, CFD, CFH, CFI, CPB2, CR1, CR2, F10, F11, F13A1, F13B, F2R, F3, F5, F7, F8, F9, FGA, FGB, FGG, KLKB1, MASP1, MASP2, MBL2, PLAT, PLAU, PLAUR, PROC, PROS1, SERPINA5, SERPINC1, SERPIND1, SERPINE1, SERPINF2, TFPI, THBD, VWF
KEGG:05130	Pathogenic Escherichia coli infection	1,61E-10	1	PRKCA, ARPC3, FYN, ABL1, NCL, ITGB1	ACTB, ACTG1, ARHGEF2, ARPC1A, ARPC1B, ARPC2, ARPC4, ARPC5, ARPC5L, CD14, CDC42, CDH1, CLDN1, CTNNB1, CTTN, EZR, HCLS1, KRT18, LOC100506658, LY96, NCK1, NCK2, RHOA, ROCK1, ROCK2, TLR4, TLR5, TUBA1A, TUBA1B, TUBA1C, TUBA3C, TUBA3D, TUBA3E, TUBA4A, TUBA8, TUBAL3, TUBB, TUBB1, TUBB2A, TUBB2B, TUBB2C, TUBB3, TUBB4, TUBB6, TUBB8, WAS, WASL, YWHAQ, YWHAZ
KEGG:04614	*Renin-angiotensin system*	6,88E-09	6	AGT, MAS1	ACE, ACE2, AGTR1, AGTR2, ANPEP, CMA1, CPA3, CTSA, CTSG, ENPEP, LNPEP, MME, NLN, REN, THOP1
KEGG:05020	Prion diseases	1,45E-10	1	NOTCH1, C9, SOD1, PRNP	BAX, C1QA, C1QB, C1QC, C5, C6, C7, C8A, C8B, C8G, CCL5, EGR1, ELK1, FYN, HSPA1A, HSPA5, IL1A, IL1B, IL6, LAMC1, LOC100506742, MAP2K1, MAP2K2, MAPK1, MAPK3, NCAM1, NCAM2, PRKACA, PRKACB, PRKACG, PRKX, STIP1

Analysis with in the CIS subgroup revealed the important pathways in the transmission of disease subtypes. MS suggestive CIS (MSs-CIS) group indicated the importance of Aldosterone regulated sodium reabsorption pathway. Single attack (SA-MS) showed the increased activation of NOD-like receptor signaling pathway and clinically definite MS (CDMS) showed shared pathways with RRMS and CIS subtypes.

Randomly generated MS group‘s pathway analysis revealed that rennin-angiotension system pathway was commonly affected in all 10 random runs for all MS groups. Complement and coagulation, prion disease, notch signaling and vitamin absorption and digestion pathways were found to be affected in most of the runs for all the MS groups as expected. Aldestrone regulated sodium absorption pathway was not found in any of the random data analysis.

## Discussion

MS is the most common neurological disorder causing disability in young adults [4], yet its cause is still unknown with an unpredictable prognosis. In this study, we investigated the proteome content of CSFs of patients representing MS clinical heterogeneity and used the benefits of bioinformatics approaches that can shed insight into the important pathophysiologic processes of the affected pathways in main subtypes of MS.

Our findings have revealed renin-angiotensin system (RAS) and complement and coagulation cascade (CCC) being common pathways shared by all MS clinical subtypes, including early, intermediate and progressive forms of MS. The involvement of CCC and RAS pathways confirm the immune related pathophysiology of MS. [8, 35, 36] An increase of AGT and MAS1 proteins production of the RAS pathway in combination with the kallikrein-kinin system appear to induce CCC pathway. Our finding of CCC pathway proteins including upregulation of Kininogen, Plasminogen, Alpha-2-Macroglobulin, SerpinE1, SerpinA1 and SerpinG1 and downregulation of F12 protein all lead to the activation of inflammation with activation of platelets, monocyte and lymphocyte cells. Alongside with the commonly identified pathways; pathways specific to the clinical subtypes of MS were also revealed. The proteasome pathway in the CIS clinical subtype (including both MSs-CIS and SA-MS) has been over activated with the increase expression of PSMB10, PSMA2, PSMA1, PSMD13, PSMB2, PSME3 proteins. This finding may point to the presence of active ubiquitin mediated proteolysis in CIS phenotype. While the vitamin digestion and absorption pathway in the PMS clinical subtype is activated by the increase in the APOA4, APOA1, SCARB1 proteins, it has been observed that the notch signaling pathway is also activated through NOTCH2, EP300, PSEN1, DTX1, JAG2 proteins, which may reflect the fact that the vitamin metabolism of MS is dysregulated and this is supportive with the recently suggested vitamin metabolism involvement in MS pathogenesis [37].

In the present study, the increase in the NOTCH2, EP300, PSEN1, DTX1, JAG2 proteins show that NOTCH signaling pathway is also upregulated in the PMS and RRMS clinical subtypes but not in CIS groups. Further detailed analysis of individual protein players of Notch, such as the NOTCH2, JAG2 and PIK proteins, shows that PMS patients appear to have higher expressed levels than RRMS types that is in accordance with the disease severity reflecting neurodegeneration level (2.2 fold, p<0,05). However RRMS clinic subtype patients also show an increase in the ACTB, KRT18, FYN, TUBA4A, NCL, CTNNB1 proteins which are a part of pathogenic *E*.*coli* infection pathway, which induce the mobilization of inflammatory cells. These findings elucidate for the first time the critical molecular pathways that are closely associated with the inflammatory and neurodegenerative mechanisms in MS. The mentioned pathways are in accordance with the current understanding that inflammatory processes are more activated in the earlier phases of the disease (CIS and RRMS clinical subtypes), more than the progressive forms.

### The importance of renin angiotensin system and complement-coagulation cascade pathways in MS pathophysiology

The role of RAS and CCC involvements in MS derives from a several indirect studies of Experimental Autoimmune Encephalomyelitis (EAE) models and directly from post-mortem tissues of MS patients [38]. Treatments with various renin inhibitors resulted with significantly ameliorated course of EAE in rats and up-regulated RAS proteins were observed in brain lesions of MS patients. Similarly *in vivo* administration of coagulation cascade inhibitors such as ACE inhibitors reduced the clinical severity EAE model, supporting the view that the blockade of the coagulation cascade would be a beneficial approach for the treatment of MS [39]. Also a recent proteomics study of MS lesion-specific proteome profiling showed a pivotal role of coagulation cascade proteins in chronic active demyelination [40].

Our study indicates the importance of RAS and CCC pathways in CSF samples of clinically different MS patients. Although each clinical subtype shows specific pathways compared to controls, all subtypes commonly showed increased RAS activity. The results of this study for the first time confirm through proteomic and bioinformatic approaches previous findings from EAE models and MS postmortem studies [38, 41, 42].

### The importance of sodium reabsorption in MS

Possible effects of salt intake on the development of neuroinflammation has recently been investigated which might link the observed increase in the incidence of multiple sclerosis (MS) and other autoimmune diseases over the past 50 years [23, 43–45]. A current study by Kleinewietfeld et al. revealed that increased dietary salt intake might represent an environmental risk factor for the development of autoimmune diseases through the induction of pathogenic TH17 cells. In this study TH17 cells from the EAE mice generated under high-salt conditions display a highly pathogenic and stable phenotype characterized by the upregulation of the pro-inflammatory cytokines GM-CSF, TNF-a and IL-2. Moreover, mice fed with a high-salt diet develop a more severe form of EAE, in line with augmented central nervous system infiltrating and peripherally induced antigen-specific TH17 cells [23]. In our study, aldosterone regulated sodium reabsorption pathway was significantly affected in CIS patients (p = 1,78E-04) compared to the control group. Pathway associated genes found in subnetworks for this pathway were, Phosphatidylinositol-4,5-Bisphosphate 3-Kinase, Catalytic Subunit Beta (PIK3C1), Phosphoinositide-3-Kinase, Regulatory Subunit 1 (Alpha) (PIK3R1), Phosphoinositide-3-Kinase, Regulatory Subunit 2 (Beta) (PIK3R2) and insulin receptor (INSR) proteins. Expression of all these proteins were increased in the CIS patients compared to control group. Our results indicate that in the CIS subtype, which represents the first clinical expression of the disease and also the initial phase of other forms, exhibited an upregulation in the reabsorption of salt pathway. Furthermore subgroup analysis of CIS patients also indicated the importance of this pathway in CIS (MSs-CIS and SA-MS) patients who had not shown conversion to RRMS in a mean follow of 30.6 months. These results may signify the importance of increased salt mechanisms in the conversion to the clinically definite MS subtype.

### The importance of vitamin metabolism in MS and its molecular basis

The epidemiological evidence suggest that exposure to sunlight may play a protective role in autoimmune diseases including MS, insulin-dependent diabetes mellitus, and rheumatoid arthritis [46]. Among many, vitamin D is the most prominent and studied one. Genetic factors linked to vitamin D receptor genes and vitamin D—binding proteins have been extensively evaluated and contradictory results have been obtained from different populations [47–49]. However CSF findings of vitamin metabolism is still missing due to the lack of specific proteomic studies. Our results revealed the increased expression of vitamin-metabolism related molecules in CSF of RRMS and CIS patients indicating the significantly increased activity of vitamin digestion and absorption pathway. Apolipoprotein family proteins that are part of the vitamin metabolism (APOA4, APOE, APOB) were significantly increased in the CSF of RRMS and CIS patients. Further subgroup analysis, comparing both CIS groups (MSs-CIS & SA-MS), with the CDMS in the 31.7 months of follow-up, showed an increased vitamin absorption and digestion related protein expression in CIS patients. ELISA studies with Vitamin D Binding Protein (VDBP) also showed the decreased VDBP level in CIS patients indicating the less amount of vitamin D metabolism in CIS subgroup (p<0.001) (S1 Fig). However, the pathogenetic and theraupetic implications of these findings need to be further explored.

### Clinical conversion of CIS to MS is regulated via specific pathways

The prognosis of patients with CIS are unpredictable, as it is not obvious whether the affected patients will experience further clinical symptoms, and then by definition, go on to be diagnosed with CDMS likely because of the activation or inhibition of different pathological mechanisms in the affected individual with CIS presentation [50, 51].

CIS group specific subgroup proteomic analysis indicated that conversion from CIS to CDMS depends on the activation of specific pathways. Non-converters are CIS-remaining-CIS show the increased expression of notch signaling, type II diabetes mellitus and aldosterone regulated sodium reabsorption pathways compared to SA-MS and RRMS groups. All three pathways are immune related pathways, which are functional in the T cell lineage commitment from common lymphoid precursor and T cell development, immune activation of Treg cells and increase in the expression of inflammatory cytokines. On the other hand, the sodium reabsorption pathway, which is also related with salt metabolism, was also activated in the MSs-CIS group remaining as such during the time course of the study. However, the SA-MS group, which has a higher probability to convert to CDMS and continues to show MRI activity and who by definition are diagnosed as early MS showed an intense inflammation possibly through the activation of the NOD-like receptor-signaling pathway. The CDMS group, showed the involvement of both neurodegenerative and inflammatory pathways, which are prion diseases and pathogenic *E*.*coli* infection pathways.

All CIS samples were diagnosed as CIS when their CSF samples were taken according to criteria of Poser [24]. These observations may have therapeutic implications for CIS patients regarding early long-term MS treatment decisions. Our results indicated the different molecular pathways are involved in CIS patients who will show further clinical and imaging disease activity. It may be concluded that SA-MS patients who do not convert to CDMS may not do so because of the upregulation of the sodium reabsorption pathway. This is a thought provoking hypothesis supportive of Haffler’s study [23] indicating the increased salt intake might represent an environmental risk factor for the development of MS through increased auto-inflammation. It is difficult to say whether the upregulation of sodium reabsorption pathway is a cause or effect of the change from CIS to MS. Our opinion at this point is that, upregulation of sodium reabsorption pathway, may induce conversion from CIS to RRMS subtype. Previous studies indicated the activation of inflammatory cells in response to higher salt intake *in vitro*. However this proposition requires further analysis using in vivo studies.

The statistical significance of the pathways is also established by randomization of the patient data. Pathway analysis of randomized data analysis also showed that aldosterone dependent sodium reabsorption pathway is not present in any of the analysis (S1 Table). This pathway is found to be significantly affected only in the analysis of CIS converting to clinically definite MS patients’ data. Other commonly found pathways in randomized MS data analysis are in agreement with the pathways in MS pathophysiology.

### Limitations of the study

To our knowledge this is the first study revealing differentially affected pathways between MS clinical phenotypes using proteomic and bioinformatic approaches, but it has its limitations too. Firstly it is already known that proteomic studies show great variations based on the individual characteristics of the samples [52, 53]. Our average global matching CVs of protein spots in the whole sample was between 22–26%, which is in the range of generally accepted values for proteome analysis of biological samples [54, 55]. Methodological approaches to overcome these technical issues are given in the methods section. Another limitation is the lack of resampling of CIS samples when they have converted to clinically definite MS or remained as CIS in the following years. This sampling would create an opportunity to observe the molecular changes in individual CSF samples in terms of affected proteins and pathways due to prospective changes in the subclinical phenotyping of CIS and MS. However resampling of CSF for a patient by using lumbar puncture is not applied because of the ethical and diagnostic criteria requirements. The identified pathways in our study include many other proteins than the ones stated here. Some of the proteins were analyzed individually using Western and/or ELISA methods, which are also given in the S1 Results, but the other pathway components also required to be investigated in order to verify their diagnostic and prognostic potentials for MS.

## Conclusion

Our results revealed common and subtype specific pathways for all MS clinical subtypes. Identified pathways are related with the inflammatory and degenerative characteristics of MS. Besides, the sodium digestion and reabsorption pathway, which is related to salt metabolism, appear to be critical in the conversion of CIS to clinically definite MS. Vitamin absorption and digestion pathway was also important in the disease subclinical forms. Our results are the first pathway oriented and proteomic based study indicating disease related molecular pathways, and results were supportive for earlier experimental and clinical molecular studies and demands further confirmative and explorative studies.

## Supporting Information

S1 Data Proteome data of our study. Protein list and their corresponding accession numbers and fold changes relative to control master gel spots were given for each individual patient samples. As it is indicated in the manuscript, our methodology is based on the comparison of the identified protein spots between individual patients gels and control gel. To this aim, we performed MALDI-TOF analysis for detection of the protein spots not for all samples, but we compared all patients gel for identified protein spots. Data is also shared via Harvard Dataverse network (doi:10.7910/DVN/28497).
(ZIP)

S1 Fig Confirmation study for vitamin D Binding protein (VDBP). ELISA targeting the VDBP was performed for all patient and control samples included in the study (65 CIS, 72 RRMS, 42 PMS and 42 control samples). The results were correlated with the 2D-PAGE studies. CSF level of VDBP protein was significantly differed in CIS and RRMS group, but not in PMS group.
(TIFF)

S1 Results Supplementary results summarize the common and shared candidate protein biomarkers in different disease subtypes. (DOCX)

S1 Table Random selection of the samples from the patient cohorts resulted with the molecular pathways in each of clinical subtypes. Numbers indicate the counts of revealed pathways in subtype. As expected Renin-angiotensin system and complement and coagulation cascades were revealed in each subtypes more than by chance. Prion disease also revealed in each disease subtypes. Aldosterone regulated sodium reabsorption pathway did not hit any of subtypes by random selection.
(DOCX)
