# Supplementary figures and images for: CSF Proteomics Identifies Specific and Shared Pathways for Multiple Sclerosis Clinical Subtypes
Source: PLoS One. 2015 May 5;10(5):e0122045. doi: 10.1371/journal.pone.0122045 (PMC4420287; doi:10.1371/journal.pone.0122045)

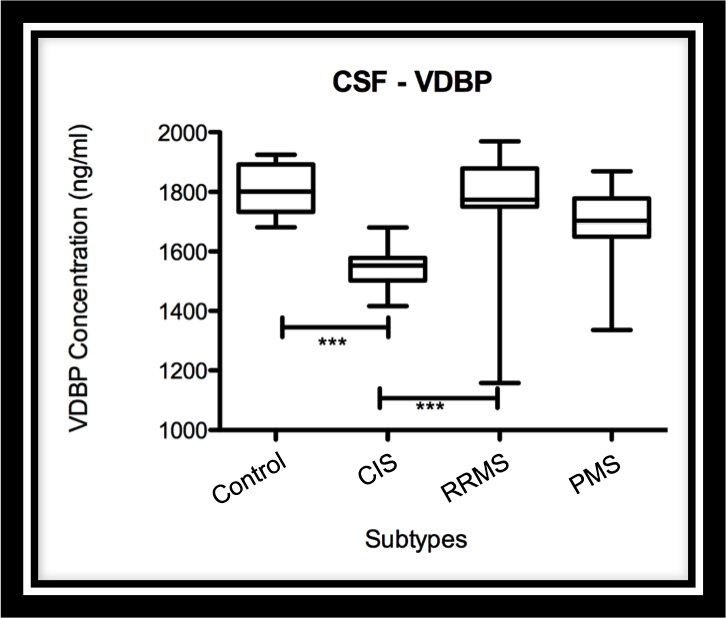

Supplement: S1 Fig — ELISA targeting the VDBP was performed for all patient and control samples included in the study (65 CIS, 72 RRMS, 42 PMS and 42 control samples). The results were correlated with the 2D-PAGE studies. CSF level of VDBP protein was significantly differed in CIS and RRMS group, but not in PMS group. (TIFF) [file pone.0122045.s002.tiff]
