# Supplementary material for: CSF Proteomics Identifies Specific and Shared Pathways for Multiple Sclerosis Clinical Subtypes
Source: PLoS One. 2015 May 5;10(5):e0122045. doi: 10.1371/journal.pone.0122045 (PMC4420287; doi:10.1371/journal.pone.0122045)
Supplement: S1 Results — (DOCX) [file pone.0122045.s003.docx]

**Supplementary Results:**

**Common protein biomarkers in all disease subtypes**

Proteomic study revealed a number of proteins that are differentially expressed in all disease clinical subtypes compared to total control groups. Among them transferrin, Chain A Alpha 1 Anti - Trypsin, Apolipoprotein A4 and Apolipoprotein E with several albumin isoforms, were abundantly observed in CSF of disease clinical subtypes.

**Subtype specific biomarkers**

Total of 151 proteins were found to be differentially expressed in the three clinical subtypes; namely CIS, RRMS and progressive MS groups. But some of them were more abundant than the rest. The differentially expression (2 fold) presence of alloalbumin was 100% in CIS, 72% in RRMS and 16% in PMS. For alpha – 2 macroglubulin presence were, 36% in CIS, 100% in RRMS and %22 in PMS, Immunoglobulin proteins G (IgG2, IgG 3, immunoglobulin light chain) proteins have been found in %48 of CIS, 54% of RRMS and 100% of PMS patients.

Some proteins showed abundance in more than one subtype. Human alpha – 1 Acid Glycoprotein have been differentially found in 100% of CIS and RRMS patients but 42% of PMS patients. Likewise prostaglandin D2 Synthase (21 kDa) found in 100% of CIS and RRMS patients but 34% of PMS patients. Superoxide dismutase was found in 82% of CIS, 26% of RRMS and CIS patients. 14-3-3 protein was found in 92% of CIS, 96% of RRMS patients and 14% of PMS patients. SPARC like protein was 18% of CIS, 92% of RRMS patients and 18% of PMS patients. Cytotoxic T Lymphocyte protein 4 was found in 100% of CIS, %76 of RRMS patients and %72 of PMS patients.

Beside the subtype specific abundant proteins, regarding all individual CSF samples of patients, comparison between total control group and disease subtypes revealed 61 different proteins in RRMS, 58 different protein in CIS and 60 proteins in PMS subtypes that are at least 2 fold differentially expressed.
