# Supplementary material for: CSF Proteomics Identifies Specific and Shared Pathways for Multiple Sclerosis Clinical Subtypes
Source: PLoS One. 2015 May 5;10(5):e0122045. doi: 10.1371/journal.pone.0122045 (PMC4420287; doi:10.1371/journal.pone.0122045)
Supplement: S1 Table — Numbers indicate the counts of revealed pathways in subtype. As expected Renin-angiotensin system and complement and coagulation cascades were revealed in each subtypes more than by chance. Prion disease also revealed in each disease subtypes. Aldosterone regulated sodium reabsorption pathway did not hit any of subtypes by random selection. (DOCX) [file pone.0122045.s004.docx]

| **PATHWAYS** | **CIS** | **PPMS** | **RRMS** |
| --- | --- | --- | --- |
| **Adherens junction** | 1 | 1 | 2 |
| **B cell receptor signalling pathway** | 2 | 1 | 1 |
| **Bacterial invasion of epithelial cells** | 2 | 1 | 1 |
| **Bladder cancer** | 0 | 1 | 0 |
| **Chronic myeloid leukemia** | 3 | 4 | 5 |
| **Citrate cycle (TCA cycle)** | 0 | 0 | 1 |
| **Colorectal Cancer** | 2 | 2 | 4 |
| **Complement and coagulation cascades** | 9 | 7 | 9 |
| **Fc epsilon RI signaling pathway** | 5 | 1 | 1 |
| **Glioma** | 0 | 1 | 0 |
| **Notch signaling pathway** | 7 | 5 | 5 |
| **Pancreatic cancer** | 0 | 2 | 1 |
| **Pathogenic Escherichia coli Infection** | 3 | 3 | 2 |
| **Prion Diseases** | 8 | 8 | 8 |
| **Prostate Cancer** | 2 | 3 | 4 |
| **Proteasome** | 0 | 1 | 0 |
| **Renin-angiotensin system** | 10 | 10 | 10 |
| **Thyroid cancer** | 2 | 1 | 3 |
| **Type II diabetes mellitus** | 1 | 1 | 1 |
| **Vitamin Digestion and Absorption** | 5 | 6 | 4 |

**Supporting Table (S2_Table):** Molecular pathways revealed by the analysis of randomly selected samples

Random selection of the samples from the patient cohorts resulted with the molecular pathways in each of clinical subtypes. Numbers indicate the counts of revealed pathways in subtype. As expected Renin-angiotensin system and complement and coagulation cascades were revealed in each subtypes more than by chance. Prion disease also revealed in each disease subtypes. Aldosterone regulated sodium reabsorption pathway did not hit any of subtypes by random selection.
